# COVID-19-Related Health Literacy of School Leaders in Hong Kong: A Cross-Sectional Study

**DOI:** 10.3390/ijerph191912790

**Published:** 2022-10-06

**Authors:** Sam S. S. Lau, Eric N. Y. Shum, Jackie O. T. Man, Ethan T. H. Cheung, Padmore Adusei Amoah, Angela Y. M. Leung, Kevin Dadaczynski, Orkan Okan

**Affiliations:** 1 Research Centre for Environment and Human Health, School of Continuing Education, Hong Kong Baptist University, Hong Kong, China; 2 Multidisciplinary Research Centre, School of Continuing Education, Hong Kong Baptist University, Hong Kong, China; 3 College of International Education, Hong Kong Baptist University, Hong Kong, China; 4 Institute of Bioresource and Agriculture, Hong Kong Baptist University, Hong Kong, China; 5 School of Graduate Studies, Department of Applied Psychology, Institute of Policy Studies, Lingnan University, Hong Kong, China; 6 School of Nursing, Hong Kong Polytechnic University, Hong Kong, China; 7 Public Health Centre Fulda, Fulda University of Applied Sciences, 36037 Fulda, Germany; 8 Center for Applied Health Science, Leuphana University Lueneburg, 21335 Lueneburg, Germany; 9 Department of Sport and Health Sciences, Technical University Munich, 80333 Munich, Germany

**Keywords:** COVID-19, school principals, school heads, health literacy, Hong Kong, vaccine hesitancy, self-endangering work behaviour, work satisfaction

## Abstract

To date, we know little about COVID-19-related health literacy among school leaders, particularly in East Asia. The present study aimed to assess the level of COVID-19-related health literacy and associated factors (vaccine hesitancy, self-endangering behaviour, and work satisfaction) among school leaders in Hong Kong. A cross-sectional study of 259 school leaders was carried out during the COVID-19 pandemic between April 2021 and February 2022. COVID-19-related health literacy using HLS-COVID-Q22, three subscales of self-endangering work behaviour scales (i.e., “extensification of work”, “intensification of work” and “quality reduction”), and two dimensions of Burnout Assessment Tool (BAT) (i.e., psychosomatic complaints and exhaustion) were used. The study employed independent sample *t*-test, ANOVA, and multilinear regression models. The findings show that more than half (53.7%) of school leaders had insufficient health literacy. Participants with insufficient health literacy scored significantly higher in the following factors: exhaustion related to work situation (*p* = 0.029), psychosomatic complaints (*p* < 0.001), attitude about vaccination (i.e., less agree with vaccination) (*p* < 0.001), level of informing on COVID-19 related information (i.e., felt less informed) (*p* < 0.001), and level of confusion about COVID-19-related information (i.e., felt more confused) (*p* < 0.001). In a linear regression model predicting attitude about coronavirus vaccination, age (β, −0.188, 95% CI, −0.024, −0.005, *p* = 0.002) and health literacy (β, −0.395, 95% CI, −0.716, −0.361, *p* < 0.001) were the negative predictors, *F*(5, 214) = 11.859, *p* < 0.001. For the linear regression model adjusted for sex and age for predicting health literacy, the model was insignificant. Despite being a highly educated group, this study reveals that one in two Hong Kong school leaders have insufficient health literacy. Inadequate health literacy was strongly associated with a negative attitude about vaccination, low information, and confusion about COVID-19-related information. Additionally, insufficient health literacy was associated with the two secondary symptoms of burnouts. The study highlights an urgent need to develop intervention programmes to promote the COVID-19-specific as well as overall health literacy of the school leaders.

## 1. Introduction

Health literacy is a key contributor to an individual’s health and is recognised as a central pillar of health promotion [1]. According to Sørensen et al. [2], health literacy is defined as “linking to literacy and entailing people’s knowledge, motivation and competences to access, understand, appraise, and apply health information in order to make judgments and take decisions in everyday life concerning healthcare, disease prevention and health promotion to maintain or improve quality of life during the life course”. Health literacy aids in exploring the sources of health information and health services, as well as discerning between reliable material and disinformation [3]. Health literacy is regarded as an asset for empowering people in disease prevention and health promotion [2]. Research showed a positive association between health literacy and health outcomes, and that health literacy closely reflects an individual’s perceived health quality [4]. People with higher health literacy have advantages in health compared with those with lower health literacy, and low health literacy is a major predictor of hospital admission [5]. Because of the COVID-19 pandemic, health literacy has been gaining increasing attention, being a health indicator of health-promoting behaviours and a critical resource for enhancing health and well-being [6], as people are required to search, select, appraise, and apply or utilise the appropriate, accurate health information for personal healthcare and infection prevention [7]. Limited health literacy has shown to be a serious public health problem, especially during the COVID-19 pandemic, but still remains underestimated in the context of public health responses [8]. A previous study showed that up to 47% of the European population is believed to have low or insufficient levels of health literacy, which is harmful to health over the life course [9]. Thus, more research is needed to assist and develop interventions in improving health literacy level of individuals worldwide, especially in times of global public health crisis.

### 1.1. Role of Schools in Promoting Health Literacy

Investing in health literacy of children and adolescents’ promises benefits for their health and well-being development over time [10,11]. Therefore, health literacy in schools must be understood as a key public health concern [12]. Schools have long been considered an essential setting to support disease prevention, health promotion, and the development of health literacy of students, teachers and administrators [10,13,14,15]. Embedding health literacy in school health promotion strategies, such as the Health Promoting School approach, is the most sustainable way to foster health literacy development early in the life-course [1,11,16]. A WHO evidence synthesis recommends that addressing health literacy in schools offers a wide range of co-benefits to society, including better health outcomes and lower healthcare costs [9]. In particular, the relational model of health literacy as provided by Parker and Ratzan [17] makes it clear that one has not only to address personal health literacy skills, but the complexities and demands of systems, including professionals, people interact with. Based on this model, the organisational health literacy approach was developed to improve environmental factors and systemic conditions of healthcare settings [18]. This concept was recently adapted to schools in order to address both agency (personal health literacy within the behavioural approach) and structure (organisational health literacy within the social approach) [19]. In contrast with the focus on personal health literacy, employing the organisational health literacy concept provides a perspective to address the health literacy of key professionals (e.g., teachers, principals, school staff, and school nurses) in a setting [14]. Educators are key to controlling and preventing the spread of COVID-19 in educational settings [20]. Research highlighted the critical role of early years services and schools in developing health literacy skills early in the life course [21]. Being role models for students in the school environment, school principals and teachers play a pivotal role in the promotion of students’ health literacy [16,22]. The organisational health literacy approach will help schools to become health literate [19], in turn promoting personal health literacy empowerment of the teachers to educate students on health decision-making skills [23,24]. Overall, schools can contribute to the achievement of public health goals in tandem with their educational commitments [21,25], and health literacy is an important tool to support this, especially if addressed in school principals and teachers. 

### 1.2. Limited Health Literacy of School Personnel 

Few studies have examined health literacy among school personnel. A recent German study of 680 school leaders indicated that 29.3% had limited health literacy [26]. With regard to teachers, a Sri Lanka study of 520 secondary school teachers showed that 31.5% had limited health literacy [27]. An Iranian study of 704 pre-service teachers demonstrated that 50.6% had either problematic or inadequate health literacy, while teachers’ limited health literacy was identified as a barrier for enhancing the health literacy of students at schools [28]. A study in Turkey of 500 school teachers showed that over 70% of the teachers had very limited or limited health literacy levels [29]. With regard to East Asian regions, a survey was conducted in Japan with 1000 educators during the COVID-19 pandemic using the European Health Literacy Survey Questionnaire (HLS-EU-Q47). The health literacy score was 33.5 ± 7.61 of 50, indicating that educators attained sufficient health literacy [20]. These findings demonstrated that the sufficiency of health literacy among school personnel varies across the world, and it may be influenced by the emergence of global public health crises. Two important questions then are: what the factors that account for the health literacy of these school workers are; and how their health literacy status potentially affects their attitudes, views and perspectives on health-related issues during the COVID-19 pandemic. 

Driven by the current COVID-19 pandemic, health literacy has been the subject of increasing attention in different occupational groups, whilst primarily focusing on healthcare workers [30] and teachers [31]. However, studies on health literacy among school leaders remain scant. Results from a recent German study showed that about three in ten school principals had a limited level of health literacy, where male principals had a significant disadvantage in health literacy [26]. Dadaczynski et al. [16] reported that principals are the key facilitator for delivery and implementation of actions on health promotion in schools, which would also be beneficial to their health literacy, especially for male principals. Health literacy of school personnel (i.e., teachers and school principals) in Hong Kong has long been ignored in the past decade. Research has tended to focus more on examining the teacher’s knowledge in delivering health education to students. For example, Cheng and Wong [32] found that the knowledge level related to hypertension was generally below average among the teachers. Thus, it is worth investigating the state of the school leaders’ health literacy and factors associated with it, especially during this large-scale global public health crisis.

### 1.3. Factors Associated with Health Literacy 

Evidence shows that there is an association between low levels of health literacy and poorer health outcomes [33,34]. Low levels of HL were found to be associated with inadequate utilisation of preventive health services, higher prevalence of chronic illness, weak intermediate disease indicators, a lack of health knowledge and increased hospitalisation rates [35,36,37]. In terms of infectious diseases, limited or poor health literacy was linked to lower adoption of protective behaviours, including vaccination and a lack of understanding of antibiotics [38]. On the other hand, health literacy was found to be positively correlated with hand hygiene, playing a role in fighting infectious diseases [39]. These studies highlighted the costs associated with insufficient health literacy and explained the importance of health literacy in the context of infectious diseases. The influential factors for the level of health literacy in the context of COVID-19 warrant further research. Recently, COVID-related health literacy among school principals was investigated in Taiwan. The findings showed that male principals with higher levels of health literacy had a lower level of vaccine hesitancy [40]. 

In terms of SARS-CoV-2 vaccination hesitancy, recent findings suggested that it was found to be associated with health literacy in the general population [41]. A study of community population in China reported that people with higher health literacy levels showed reduced vaccine hesitancy [42]. People with better health literacy and vaccine literacy were suggested to have lower vaccine hesitancy [43]. However, whilst health literacy seems to be influenced by few factors, such as age, country and types of vaccine, a systematic review suggested that the relationship between health literacy and influencing factors remains unclear [44]. In Hong Kong, the intention of getting vaccinated was low in the Hong Kong general population in late 2020 [45]. Younger adults in Hong Kong tend to have higher vaccination hesitancy, where mistrust in government was found to be associated with higher vaccination hesitancy [45]. However, empirical studies of the effect of health literacy on vaccination hesitancy have been rarely discussed in the context of Hong Kong, especially among school personnel. Thus, the relationship between health literacy and vaccine hesitancy warrants our investigation, especially among the school personnel in Hong Kong.

### 1.4. Health Literacy in the Context of Work

Making informed health decisions might be helpful in occupational and health contexts, such as when addressing issues like recuperating from illness, missing work due to illness, and reintegrating back into society and the workforce [46]. According to Güner et al. [47], a lack of health literacy may be one of the obstacles preventing people from fully understanding and benefiting from occupational training. Six of their suggested health literacy structural model’s elements, including self-perception, proactive attitude to health, self-control, self-regulation and communication and collaboration, were linked to job performance in a prior research of young adults who were employed [48]. Despite the fact that prior research in Iran found no connection between work stress and health literacy, it is doubtless that people were experiencing an uncontrollable stressful situation during COVID-19 [49]. Certain occupational groups, such as frontline nurses and teachers worldwide, felt more stressed than they did before the pandemic [50,51]. Due to the school closures during the pandemic, a lot of strain and stress was placed on the educational system, forcing it to switch to a digital teaching paradigm, adapt to different technology tools and improve its computing abilities. As expected, this change presented a number of difficulties, particularly for teaching professionals who had to deal with the strain caused by the COVID-19 illness itself, as well as the technological difficulties associated with the creation of the academic material, in order to continue immersing the students in order to provide the desired academic experience [52]. In the face of increased workload and strong demand of self-management, scholars proposed that self-endangering work behaviour will be performed as coping reactions, such as work intensification, extensification and quality reduction [53]. However, performing these self-endangering work behaviours may be detrimental to one’s health and long-term ability to work [53]. Work overloads or stress overloads may cause burnout syndrome, such as psychosomatic complaints and feeling of exhaustion, as well as the emergence of minor to severe mental illnesses, such as depression and anxiety [52]. 

Despite mounting research examining the levels of health literacy, few researchers have investigated the health literacy levels of school leaders. To the best of our knowledge, no reports have examined the health literacy of school leaders in Hong Kong during the COVID-19 pandemic. Moreover, there are no reports addressing the associated factors in relation to the level of health literacy. Therefore, the aims of this study were to (a) assess the COVID-19-related health literacy among school leaders in Hong Kong, (b) examine the associated factors in affecting the health literacy of school leaders, (c) examine the differences in health literacy in relation to different demographic variables, (d) examine the differences in health literacy in relation to the level of COVID-related information received, and (e) examine the effect of health literacy on COVID-19 vaccine hesitancy among school leaders in Hong Kong.

## 2. Materials and Methods

### 2.1. Study Design, Study Population and Data Collection

This study is part of the COVID-19 Health Literacy Network (https://COVID-hl.eu, accessed on 17 September 2022), a global research network involving over 150 researchers from 75 countries focusing on health literacy research in relation to COVID-19. We used a descriptive cross-sectional design. As part of a larger study which aimed to examine the work and health situation of the school community in Hong Kong, this study was conducted between April 2021 and February 2022 during the period of strict social distancing measures implemented in Hong Kong in a zero-COVID policy context. The English-language survey was adapted from Dadaczynski et al. [54] and translated into the traditional Chinese version, taking into consideration the conceptual, cultural and linguistic contexts in Hong Kong. A pilot study of the translated version was conducted with 8 individuals working in the education sectors. Feedback and disagreement were considered by the first author. The translated version was further reviewed and rephrased between the first, third, fourth and sixth authors until a satisfactory version was reached. Permission to conduct the research was obtained from the Research Ethics Committee of Hong Kong Baptist University (REC/20-21/0465). Informed consent was obtained from all participants. The study included adult participants (18+ years) only. The survey was anonymous and administered electronically using the Qualtrics platform, and participation was voluntary and confidential. Participants were allowed to terminate the survey at any time. The survey took 15–20 min to complete. 

The target participants were school leaders, including principals, vice principals, assistant principals and other leaders (e.g., members of the school management boards, Dean of Admission) in primary, secondary and special schools in Hong Kong. First, we sent email invitations to the online survey in both English and Chinese to the principals of 1130 schools registered with the Education Bureau of the government in Hong Kong, including 561 primary schools, 477 secondary schools and 36 special schools. We also contacted our school networks by phone and mailed hardcopy questionnaires to 243 school principals after obtaining verbal consent. In addition, to expedite the participant recruitment, principals were recruited via authors’ personal networks conducted though social media platforms (i.e., WhatsApp). We also recommended that respondents invite eligible participants to participate. The final sample consisted of 259 school leaders. According to the G Power software version 3.1.9.7 for Windows, the required sample size was calculated as 213, with an effect size of 0.1, type I error of 0.05, power of 0.95 and 18 potential predictors in multiple linear regressions. 

### 2.2. Measurements

#### 2.2.1. COVID-19 Health Literacy

We used a self-reported 22-item corona-specific health literacy scale (HLS-COVID-Q22), with the Cronbach alpha value of 0.94, to assess participants’ ability to comprehend and apply health information related to COVID-19 [55,56]. Participants rated how ease of access, understanding, appraisal and application of the information related to health in the context of the COVID-19 pandemic, on a 4-point scale from 1 (very difficult) to 4 (very easy). The mean score of the response values was calculated. A higher total score indicates a higher level of health literacy. A mean score of lower than or equal to 2.5 indicates inadequate health literacy. A mean score of above 2.5, but below 3 indicates problematic health literacy. A mean score of equal to 3 or above indicates sufficient health literacy. The Cronbach alpha value of the present study was 0.955. 

#### 2.2.2. Physical and Mental Health-Related Factors

The exhaustion related to work situation was assessed using the “exhaustion” subscale of the Burnout Assessment Tool (BAT). Participants were asked to rate how often the statements applied to them on a five-point scale, from 1 (never) to 5 (always). The total exhaustion scores were calculated by the sum score of the 3 items and divided by the number of items. A higher score shows stronger exhaustion. The “Exhaustion” subscale has satisfactory reliability (Cronbach α = 0.85–0.87) [57]. Self-reported psychological discomfort was measured by the “Psychosomatic complaints” symptoms of BAT. The scale has 5 items, such as “I suffer from palpitations or chest pain”, that are rated on a 5-point scale, from 1 (never) to 5 (always). The total score is calculated by adding the scores of the items and dividing by the number of items, ranging from 1 to 5. A higher score indicates more psychosomatic complaints. The total score of “exhaustion” and “psychosomatic complaints” can be classified into 4 sectors: low, average, high and very high [57]. For a total score between 3.00 to 3.99, it was classified as “high”. For a total score equal to or above 4, it was classified as “very high”.

#### 2.2.3. Self-Endangering Behaviour

Self-endangering work behaviour is a coping mechanism that buffers diminished well-being in response to heavy workloads and a lot of pressure to organise oneself [53]. Three subscales of the self-reported self-endangering work behaviour scales [58] were adopted to measure participants’ self-endangering behaviours. The subscales used in the current study included “Extensification of work”, “Intensification of work” and “Quality reduction”. The “Extensification of work” subscale consisted of six items, including “… give up leisure activities in favour of work”, whilst the “Intensification of work” subscale and the “Quality reduction” subscale both consisted of three items, including “…you find burdensome?”, and “…be satisfied even with a less good work result than you would normally be?”. Responses to items were scored on a 5-point scale, from 1 (never/very rarely) to 5 (very often). The score for each subscale was calculated by summing the item scores in the respective subcategories and dividing it by the number of items. A higher score represents a higher level of extensification of work, intensification or quality reduction, respectively. The reliability of the extensification of work subscale was very good (Cronbach α = 0.81) and excellent for the intensification of work subscale (Cronbach α = 0.93) [58], whereas in this study, the Cronbach alpha values of the three subscales were 0.891, 0.892 and 0.721, respectively. 

#### 2.2.4. Health Information in the Context of the COVID-19 Pandemic 

We measured how well informed participants feel about the coronavirus or the corona pandemic with a 5-point scale, ranging from 1 (very informed) to 5 (insufficiently informed) [55]. The feeling of confusion about COVID-19 information was also measured using a four-point rating scale, ranging from 1 (not at all confused) to 4 (very confused) [55]. 

#### 2.2.5. Attitudes towards Vaccination

The willingness to get vaccinated against COVID-19 if offered was also assessed by rating on a 6-point scale, ranging from 1 (yes, certainly) to 5 (certainly not) and 6 (I am already vaccinated). A self-reported survey measuring attitudes towards vaccination was adopted. It consists of 5 items, including “vaccinations are important to protect me and my family.”. The item “Vaccination is in line with my religious beliefs” was separated into 2 items: “Vaccination is compatible with my attitudes”, and “Vaccination is compatible with my religious beliefs.” Participants were asked to rate how much they agree with the statements, by rating on a 4-point scale, ranging from 1 (totally agree) to 4 (not agreeing at all). The mean score of the items was calculated and used. The Cronbach alpha value of the present study was 0.882. 

#### 2.2.6. Control Variables: Demographic and Work-Related Characteristics 

To collect information about the demographic and work-related characteristics of the participants, closed questions were used, which revealed information regarding gender (female, male), age, school types (primary school, secondary schools, special schools), position at school (principal, vice principal, assistant principal, member of the school management committee, leadership team).

### 2.3. Statistical Analysis

Data analysis was undertaken using SPSS Version 27.0. Continuous variables were described using mean (M) and standard deviation (*SD*), whereas categorical variables with number (*n*) and percentage (%). Independent sample *t*-tests were used to compare the difference in health literacy among groups, such as gender, positions at school and type of schools, while associated factors of COVID-19-related health literacy were tested using bivariate and multivariate linear regression models, including the level of informing on COVID-related information, level of confusion on COVID-related information, self-endangering work behaviour (i.e., “extensification of work”, “intensification of work” and “quality reduction”), work satisfaction and secondary symptoms of burnout (i.e., “exhaustion related to work situation” and “psychosomatic complaints”). Health literacy was used as a dependent variable for the bivariate analysis to detect differences with regard to demographic and work characteristics, but as an independent variable as a predictor for health outcomes and vaccine attitudes and behaviour. Separate multivariate linear regression models were used to investigate the effect of health literacy on vaccine attitude and perceived general health, respectively. Multicollinearity was ruled out in the analyses. For the multiple regression model developed, the F-test was used to validate the significance of the model. A *p*-value of less than 0.05 is considered statistically significant. 

## 3. Results

### 3.1. Participants’ Demographic and Work Characteristics, Self-Endangering Behaviours and Health Information and Promotion 

The sample was composed of 259 responses from school leaders (Table 1). The mean age of the participants was 48.6 ± 7.48 (range: 22–68 years), with slightly more men (56.4%) than women. The majority were school leaders from secondary schools (67.3%), followed by primary schools (25.7%) and special schools (7.0%). Most were school principals and heads of their school (81.8%). Referring to self-endangering behaviours, the means of extensification of work, intensification of work and quality reduction were 3.61 ± 0.75, 3.07 ± 0.85 and 2.51 ± 0.76, respectively. Concerning health information in the context of the COVID-19 pandemic, over half of the participants (52.5%) were satisfied with the level of information on COVID-19 or pandemic-related information: more than two-fifths (44.0%) of the participants were well informed or very well informed. The majority of the participants (88.3%) were either not at all confused or a little confused with information on coronavirus. Over half of the participants (54.8%) were either certain or likely to get vaccinated against COVID-19, while 34.0% were already vaccinated. By using a median split, more than half of the participants (61.8%) reported a high level of implementation of health promotion activities at school, while 38.2% reported a lower implementation status. The majority of schools (80.5%) have not participated in the “Health Promoting School Programme” of the Hong Kong SAR (HKSAR) Government. Only 10.5% of schools joined the programme for 3 or more years. For the usage of a specific media literacy curriculum or action plan that regulates the use of digital media, more than half of the participants (51.2%) reported that plans were not in place at their schools, 37.7% of participants reported that the plans were implemented at schools based on Information Literacy for Hong Kong Students from the HKSAR’s Education Bureau, and the rest was based on Curriculum Resources about the COVID-19 from the HKSAR’s Education Bureau. Moreover, the mean of the sufficiency of digital media equipment was 1.99 ± 0.69, while the overall rating of the digital media equipment and infrastructure at school was 2.90 ± 1.13.

### 3.2. Health Literacy

Table 2 shows COVID-19-specific health literacy mean scores in terms of participant characteristics. The mean score was 3.09 (*SD* = 0.42). A total of 46.3% of the participants was found to have sufficient health literacy, 47.5% had problematic health literacy and 6.2% showed inadequate health literacy. For individuals classified with sufficient health literacy, the mean health literacy score was 3.42 (*SD* = 0.31), whereas individuals with problematic or inadequate health literacy presented with a mean score of 2.88 (*SD* = 0.14) and 2.21 (*SD* = 0.36), respectively. To minimise the uneven effect of sample sizes, the present study has further classified health literacy level into dichotomised groups: sufficient health literacy (*n* = 138) and insufficient health literacy (problematic + inadequate health literacy) (*n* = 121) for further analyses. The findings showed that health literacy varied between subgroups according to how well informed participants were of the COVID-19-related information and how confused they were about COVID-19-related information within the sample population. Participants who were well or very well informed on COVID-19-related information had higher health literacy levels (3.19 ± 0.46) compared to participants who were insufficiently, poorly or acceptably informed on COVID-19-related information (3.00 ± 0.37), *t*(255) = 3.528, *p* < 0.001. Participants who felt not at all or a little confused about COVID-19-related information had higher health literacy levels (3.12 ± 0.40) compared to participants who felt quite confused or very confused about COVID-19-related information (2.90 ± 0.54), *t*(255) = 2.73, *p* = 0.002. 

To compare the difference in the scores among work-related factors (i.e., self-endangering behaviours) between each pair of groups, secondary symptoms of work-related burnout) and attitude about vaccination between the dichotomised groups, independent *t*-tests were used (Table 3). Respondents with insufficient health literacy level (M = 3.24, *SD* = 0.80) scored significantly higher in “exhaustion related to work situation” of the BAT than those with sufficient health literacy (M = 3.00, *SD* = 0.89), *t*(255) = 0.493, *p* = 0.029. For another dimension of BAT, principals with insufficient health literacy level (M = 2.46, *SD* = 0.68) scored significantly higher in “psychosomatic complaints” than those with sufficient health literacy (M = 2.17, *SD* = 0.63), *t*(253) = 3.56, *p* < 0.001. Regarding attitude towards vaccination, participants with insufficient health literacy level (M = 1.94, *SD* = 0.55) scored significantly higher in attitude about vaccination (i.e., less agreement with vaccination) than those with sufficient health literacy (M = 1.54, *SD* = 0.47), *t*(242) = 6.08, *p* < 0.001.

In addition, participants with insufficient health literacy (M = 2.68, *SD* = 0.68) scored higher than those with sufficient health literacy (M = 2.30, *SD* = 0.67) in level of information about COVID-19-related information (i.e., felt less informed), *t*(255) = 4.467, *p* < 0.001. Participants with insufficient health literacy (M = 1.99, *SD* = 0.61) scored higher than those with sufficient health literacy (M = 1.68, *SD* = 0.64) in level of confusion about COVID-19-related information (i.e., they felt more confused), *t*(252) = 3.964, *p* < 0.001. 

Table 4 shows the results of a multilinear regression model for predicting health literacy by work-related factors (i.e., self-endangering behaviours, secondary symptoms of work-related burnout). The model was adjusted for age and sex. The results of the regression indicated an insignificant model; *F*(7, 218) = 1.903, *p* = 0.07, with an R2 of 0.058.

When the multiple linear regression model was adjusted for age and sex to predict attitudes towards the vaccine (Table 5), a higher score in attitude about the vaccine (less agreeable to vaccination) is associated with younger age (β, −0.188, 95% CI, −0.024, −0.005, *p* = 0.002) and lower level of COVID-19-related health literacy (β, −0.395, 95% CI, −0.716, −0.361, *p* < 0.001); *F*(5, 214) = 11.859, *p* < 0.001 with an R2 of 0.217. 

## 4. Discussion

The present study aimed to investigate Hong Kong school leaders’ COVID-19-related health literacy, their working conditions and aspects of health information, all within the context of the COVID-19 pandemic. To the best of our knowledge, this is the first health literacy study to explore COVID-19-related health literacy in school leaders in Hong Kong. We found that more than half (53.7%) of the school leaders in Hong Kong had insufficient health literacy, including 47.5% problematic health literacy and 6.2% inadequate health literacy, and only 46.3% had sufficient health literacy. The mean health literacy score of this study (3.09 ± 0.42) was comparable to a recent study in Taiwan using the same tool (3.2 ± 0.4 [40]). Compared with the general population in Hong Kong with a mean score of health literacy on COVID-19 of 38.35 over 56 measured by items extracted from a questionnaire created by the World Health Organisation (WHO) Risk Communication and Community Engagement (RCCE) Action Plan Guidance for COVID-19 preparedness and response [59,60], the participants in the study showed slightly higher health literacy with regard to COVID-19 than the general population in Hong Kong. This can be explained by the higher education attainment of the school leaders, as it was reported in previous studies that a higher level of health literacy was positively associated with individuals’ educational attainment and cognitive skills [61,62]. Given the leading role of school leaders within their school community (e.g., on wellbeing and job satisfaction of the employees [63,64]), the need to further enhance the health literacy of school leaders is warranted. It is also important to note that strengthening the health literacy of school leaders is not only essential to their health and improving their healthy behaviours, but also has beneficial effects on the health of the school teachers and staff through health-oriented leadership behaviour [65,66]. 

In this study, the results showed no significant differences in health literacy levels between male and female participants. This finding is inconsistent with previous studies. Male school leaders in Germany had a higher percentage of limited health literacy than their female counterparts [67]. In Taiwan, female school principals had a higher level of health literacy compared to males [40]. Other studies showed mixed gender effects on the health literacy of the general population. Some studies showed that women tend to have a higher level of health literacy than men [61,62,68] reported females tend to have a higher level of literacy in learning about medical forms, instructions on medicine bottles, and written information from their healthcare provider than males. However, large-scale local research showed that no significant gender differences were found across health literacy domains [50]. It is noted that the gender effects on health literacy may vary according to the time, context and even the place. Additionally, the studies used different measures of health literacy and had different samples. Future systematic reviews may be needed to capture a more comprehensive picture of the gender effects of health literacy of universal target groups. 

Our results showed that participants with sufficient health literacy would have a higher level of understanding of COVID-19-related information compared with those who had insufficient health literacy. This finding was consistent with a previous study conducted in Germany, where inadequate and problematic health literacy levels were more prevalent in people who reported themselves as “not so well informed” or “not well informed at all” [56]. COVID-19-related health literacy is related to the ease of access, understanding, appraising and applying information related to health in the context of the COVID-19 pandemic. This could be explained by advancement in technology; people have much easier access to information related to COVID-19. This was also echoed by a previous study, which found that the enhancement of information literacy skills of an individual could improve their health literacy [69]. Thus, our finding suggested that an increase in the level of understanding of COVID-19-related information among school leaders might improve their health literacy. In alignment with the finding of Okan et al. [56], the present study also found that participants who had sufficient health literacy tend to feel less confused about COVID-19-related information than those who had insufficient health literacy. According to the integrated model of health literacy [14], the four core competencies contributing to health literacy were the ability to access, understand, apply, and appraise health information. Each of these competencies may deeply determine the extent of confusion perceived by each individual. A previous study found that it is more challenging for people with limited health literacy to understand and apply health information [70]. Previous studies also revealed that people with lower health literacy were associated with a lower likelihood of accessing medical websites for health information and a higher likelihood of utilising social media, television, blogs and celebrity websites [71]. Thus, people with lower health literacy may have a higher chance of receiving inaccurate health information. However, as most of our participants were not at all or a little confused about COVID-19-related information, the level of confusion about COVID-19-related information affecting participants’ health literacy may not be conclusive. In light of misinformation and fake news in the context of COVID-19, the infodemic knowledge may cause psychological disturbance and panic, fear, depression and fatigue [72]. Policy makers should develop programmes to promote school leaders’ health literacy and select appropriate, reliable sources for accessing health information. 

In our study, results showed that higher scores in COVID-19-related health literacy was associated with a more positive attitude about vaccination. This finding aligns with research from Taiwan which shows the strong association of higher coronavirus-related health literacy and lower vaccine hesitancy [40], and also with another study conducted in Vietnam [73]. A local study by Luk et al. [74] also found that vaccine hesitancy was strongly associated with inadequate knowledge of SARS-CoV-2 transmission. Research suggested that people in Hong Kong were most concerned about vaccines’ efficacy and severe side effects when choosing a vaccine [45]. For the timing of COVID-19 vaccination, people from Hong Kong tend to wait for clarification about the vaccines’ effectiveness and safety [45,75]. As reported by Yu et al. [45], general trust toward the government is a positively associated factor of COVID-19 vaccination. Similar findings were reported by previous studies in other regions, such as Brazil and Poland [76], which found that vaccine hesitancy could be affected by mistrust of governments during the pandemic. The intentions behind COVID-19 vaccination could also be undermined by belief in conspiracy theories [77]. The perceived risk and effectiveness of the vaccine in addition to trust in the government and health authorities were found to be associated with vaccination intention among university students [78]. The same study revealed that “confidence” and “collective responsibility” were two crucial dimensions in the 5C model in explaining the vaccination intention of university students. Therefore, with an aim of increasing the intention of school personnel to have the COVID-19 vaccination, possible methods may include enhancing individuals’ health literacy and knowledge of COVID-19 transmission, clarifying information about vaccines’ efficacy and safety, promoting government’s trust and resolving conspiracies surrounding the COVID-19 vaccine. Policy makers should target promotion of precise knowledge (i.e., potential risks and effectiveness of the vaccine) to school leaders.

In terms of age, in line with the findings of a local study by Luk et al. [74], older age was associated with lower odds of coronavirus vaccine hesitancy. However, it does not seem to be a global phenomenon. According to a recent systematic review of 15 articles related to vaccine hesitancy in COVID-19, low age was associated with a lower willingness to receive vaccination [79]. Another previous large-scale study of the general population in the UK also revealed that younger age groups tend to have a higher level of COVID-19 vaccine hesitancy [80]. Thus, the effect of age on coronavirus vaccine hesitancy may differ around the world and in various groups. However, there is growing evidence that higher-level COVID-19-related health literacy, or health literacy in general, could predict the attitude toward vaccination and willingness to receive vaccination [40]. Thus, more attention should be placed on the younger age groups, regardless of the school personnel or the general population in Hong Kong.

No significant difference was found in the frequencies of performing self-endangering work behaviour, including work intensification, work extensification and quality reduction. According to previous literature, self-endangering work behaviour can be seen as a coping reaction in response to high work demands [53] and is used by employees as an active and problem-focused approach to achieving tough goals and very demanding work requirements [81]. Baeriswyl et al. [81] suggested that measuring self-endangering work behaviour could be a starting point for exploring health promotion in schools or occupational settings, but this is not the case in the current study. In contrast, the current study found that school leaders with insufficient health literacy have a higher frequency of secondary burnout symptoms, including exhaustion related to work situations and psychosomatic complaints. In the field of organisational psychology, burnout has long been a well-used indicator of poor mental health [82]. Therefore, our results indirectly indicated that school leaders with insufficient health literacy tend to have poorer mental health than those with sufficient health literacy. A previous study comparing the mental health and physical health literacy of individuals showed that participants generally had better recognition of physical problems than those of mental health disorders [83]. However, the association between mental health and physical health literacy were rarely discussed. Future studies are needed to examine the effect of physical health literacy on one’s mental health.

### Limitations and Future Research 

The research presented has several limitations that should be addressed through future studies. First, our data were based on a self-report questionnaire by school leaders, and therefore may be subject to common method bias [84]. Second, like most of the existing literature on health literacy, only cross-sectional data were collected in the present study, which may not be sufficient to support the causal relationship between health literacy and other variables. Future longitudinal studies and qualitative research are warranted to further clarify the interplay between health literacy and other variables. Third, the study adopted a convenience sampling approach, so the study population was not representative of all school principals and leaders in Hong Kong. Another limitation is the extended data collection period of 11 months of the study, which covered the third and fourth waves of the COVID-19 pandemic in Hong Kong, representing the transition from half-day face-to-face teaching to complete suspension of face-to-face classes in favour of online teaching. Thus, the workload changes during the COVID-19 pandemic may have been underestimated. Lastly, most of the data were collected in the early stage of the Hong Kong vaccination programme, when vaccination pass arrangements were not implemented. Therefore, the vaccination attitude and willingness may not be transferred to the current situation of Hong Kong. Despite these limitations, this current research adds significantly to the existing literature regarding the health literacy of school leaders during the COVID-19 pandemic. 

## 5. Conclusions

Despite being a highly educated group, one in two Hong Kong school leaders have insufficient health literacy. Our study fills the gap of health literacy literature of school leaders in East Asia, especially in China during the COVID-19 pandemic. The study highlights an urgent need to develop health educational programmes to promote and advocate the COVID-19-specific as well as overall health literacy of school leaders. The study also shows the need for organisational policies and strategies (e.g., organisational management [85]) focused on strengthening the health literacy of school leaders and illustrates the possible factors in improving their health literacy in school settings in Hong Kong. Organisational strategies, such as introducing matrix organisational structure and management through various objectives [53], may be considered by school management boards. 

## Figures and Tables

**Table 1:** School leaders’ characteristics, their works, perceived stress, health, COVID-19-related information and health literacy, sense of coherence and well-being (*n* = 259).

Variables		Participants, *n* (%)
Gender	Male	146 (56.6)
	Female	112 (43.4)
Type of School	Primary School	66 (25.7)
	Secondary School	173 (67.3)
	Special School	18 (7.0)
Position at School	School Principal/Head of School	210 (81.8)
	Others #	49 (19.2)
Self-endangering behaviours, mean ± SD	Extensification of work	3.61 ± 0.75
	Intensification of work	3.07 ± 0.85
	Quality reduction	2.51 ± 0.76
Level of informing on COVID-19 or pandemic related information	Well or very well informed	114 (44.0)
	Insufficient/poorly/acceptably informed	145 (56.0)
Level of confusion due to COVID-19-related information	Not at all/a little confused	226 (88.3)
	Quite confused/very confused	30 (11.7)
Coronavirus vaccination readiness	Certainly/likely	142 (54.8)
	Maybe	20 (7.7)
	Certainly not/unlikely	9 (3.5)
	Already vaccinated	88 (34.0)
Attitudes about vaccination, mean ± SD		1.75 ± 0.55
Exhaustion related to work situation	Very high	82 (31.7)
	High	80 (30.9)
	Average	88 (34.0)
	Low	9 (3.5)
Psychosomatic complaints	Very high	12 (4.7)
	High	54 (21.1)
	Average	146 (57.0)
	Low	44 (17.2)

# e.g., Member of the school management committee/Incorporated Management Committee, Dean of Admissions, Director of Learning Support, Director of Operations

**Table 2:** Health literacy levels and mean scores by participant characteristics using *t*-tests and ANOVA.

Variables	Health Literacy Level [%]		Health Literacy Scores	
	Insufficient	Sufficient	Mean (*SD*)	*p*
All samples	53.7	46.3	3.09 (0.42)	*-*
Gender				
Female (*n* = 112; 43.3%)	54.5	45.5	3.09 (0.38)	0.589
Male (*n* = 146; 56.6%)	52.1	47.9	3.10 (0.42)	
School Types				
Regular School (*n* = 241; 93.1%)	53.1	46.9	3.10 (0.47)	0.280
Special School (*n* = 18; 6.9%)	55.6	44.4	2.98 (0.60)	
Positions at School				
Principals (*n* = 103; 39.8%;)	52.4	47.6	3.10 (0.45)	0.965
School vice principals (*n* = 85; 32.8%)	54.8	45.2	3.08 (0.35)	
School assistant principals/Members of the school management committee/Leadership team (*n* = 70; 27.4%)	54.3	54.7	3.09 (0.46)	
Level of informing on COVID-19-related information				
Well or very well informed (*n* = 114; 44.0%)	39.5	60.5	3.19 (0.46)	**<0.001**
Insufficient/poorly/acceptably informed (*n* = 145; 56.0%)	65.0	35.0	3.00 (0.37)	
Level of confusion due to COVID-19-related information				
Not at all/a little confused **(***n* = 226; 87.3%)	52.2	47.8	3.12 (0.40)	**0.002**
Quite confused/very confused (*n* = 33; 12.7%)	70.0	30.0	2.90 (0.54)	

Bold figures indicate *p* < 0.05.

**Table 3:** Score difference in self-endangering behaviours, secondary symptoms of burnout and attitude about vaccination based on the health literacy levels using independent *t*-test.

Health Literacy Level	Extensification of Work (SEB)			Intensification of Work (SEB)			Quality Reduction (SEB)		
	Mean (*SD*)	Mean diff.	*p*	Mean (*SD*)	Mean diff.	*p*	Mean (*SD*)	Mean diff.	*p*
Sufficient	3.63 (0.74)	−0.0295	0.758	2.98 (0.91)	0.174	0.103	2.43 (0.80)	0.135	0.128
Insufficient	3.59 (0.78)			3.15 (0.79)			2.57 (0.61)		
	**Exhaustion related to work situation (BAT)**			**Psychosomatic complaints (BAT)**			**Attitudes about vaccination**		
	Mean (*SD*)	Mean diff.	***p***	Mean (*SD*)	Mean diff.	***p***	Mean (*SD*)	Mean diff.	***p***
Sufficient	3.00 (0.89)	0.238	**0.029**	2.17 (0.63)	0.292	**<0.001**	1.54 (0.47)	0.400	**<0.001**
Insufficient	3.24 (0.80)			2.46 (0.68)			1.94 (0.55)		
	**Level of informing on COVID-19-related information**			**Level of confusion due to COVID-19-related information**			-		
	Mean (*SD*)	Mean diff.	***p***	Mean (*SD*)	Mean diff.	***p***			
Sufficient	2.30 (0.67)	0.379	**<0.001**	1.68 (0.64)	0.312	**<0.001**			
Insufficient	2.68 (0.68)			1.99 (0.61)					

Bold figures indicate *p* < 0.05.

**Table 4:** Factors associated with COVID-19-related health literacy score by multiple linear regression analyses #.

Variables	B(95%CI)	β	*p*
Age	0.002 (−0.006, 009)	0.030	0.654
Sex	−0.030 (−0.141, 081)	−0.036	0.595
Intensification of work (SEB)	−0.088 (−0.192, 0.016)	−0.180	0.095
Extensification of work (SEB)	0.153 (0.039, 0.231)	0.247	**0.006**
Quality reduction (SEB)	0.003 (−0.080, 0.086)	0.005	0.939
Exhaustion related to work situation	0.003 (−0.084, 0.091)	0.007	0.940
Psychosomatic complaints	−0.078 (−0.175, 0.020)	−0.124	0.118

Bold figure indicates *p* < 0.05. # The model was adjusted for age and sex.

**Table 5:** Associated factors of attitude about vaccine via multiple linear regression analyses #.

Variables	B(95%CI)	β	*p*
Age	−0.015 (−0.024, −0.005)	−0.188	**0.002**
Sex	0.077 (−0.063, 0.218)	0.067	0.278
COVID-19-related health literacy	−0.538 (−0.716, −0.361)	−0.395	**<0.001**
Level of understanding of COVID-19-related information	−0.006 (−0.112, 0.101)	−0.007	0.913
Level of confusion due to COVID-19-related information	0.051 (−0.063, 164)	0.058	0.381

Bold figures indicate *p* < 0.05. # The model was adjusted for age and sex.

## Data Availability

The data of this study are available from the corresponding author upon reasonable request.
